# Ki-67/MKI67 as a Predictive Biomarker for Clinical Outcome in Gastric Cancer Patients: an Updated Meta-analysis and Systematic Review involving 53 Studies and 7078 Patients

**DOI:** 10.7150/jca.30074

**Published:** 2019-08-29

**Authors:** Dan-dan Xiong, Chu-mei Zeng, Ling Jiang, Dian-zhong Luo, Gang Chen

**Affiliations:** Department of Pathology, First Affiliated Hospital of Guangxi Medical University, Nanning, Guangxi Zhuang Autonomous Region 530021, China

**Keywords:** gastric cancer, Ki-67/MKI67, prognosis, clinical value

## Abstract

Gastric cancer (GC) threatens human health worldwide and we performed this meta-analysis to evaluate the clinical value of Ki-67/MKI67 in patients with GC. The combined hazard ratio (HR), odds ratio (OR) and 95% confidence interval (95% CI) were calculated to assess the relationships of Ki-67/MKI67 expression with prognoses and clinicopathological characteristics. Genes co-expressed with MKI67 were collected for Gene Ontology (GO), Kyoto Encyclopedia of Genes and Genomes (KEGG) pathway and protein-protein interaction (PPI) network analyses. In total, 53 studies with 7078 patients were included in this study. The pooled HRs indicated that an elevated expression of Ki-67/MKI67 predicted an unfavorable overall survival (HR: 1.54, 95% CI: 1.33-1.78, *P*<0.0001) and disease-free survival (HR: 2.28, 95% CI: 1.43-3.64, *P*<0.0001) in GC patients. Additionally, in patients with advanced GC, a high Ki-67/MKI67 expression was also significantly connected with OS (HR: 1.37, 95% CI: 1.18-1.60, *P*<0.0001). The combined ORs showed that Ki-67/MKI67 expression was related to TNM stage (stage III/IV *versus* stage I/II: OR=1.93, 95% CI=1.34-2.78, *P*<0.0001), tumor differentiation (poor *versus* well/moderate: OR=1.94, 95% CI=1.32-2.85, *P*=0.001), lymph node metastasis (yes *versus* no: OR=1.67, 95% CI=1.23-2.25, *P*=0.001), distant metastasis (yes *versus* no: OR=1.67, 95% CI=1.24-2.26, *P*=0.001) and tumor invasion depth (T3/T4 *versus* T_is_/T1/T2: OR=1.98, 95% CI=1.60-2.44, *P*<0.0001). The results of GO, KEGG pathway and PPI network analyses indicated that Ki-67/MKI67 may be involved in the development of GC via influencing P53 signaling pathway. Ki-67/MKI67 could be a potential indicator to predict the prognosis of patients with GC and identify high-risk cases. Detecting Ki-67/MKI67 expression in clinic may be helpful in optimizing individual treatment and further improving the survival expectancy of patients with GC.

## Background

Gastric cancer (GC) is one of the most common and aggressive malignancies worldwide according to the World Health Organization 1. In the US, about 28,000 new cases and 10,960 deaths occurred in 2017 2. While in China, GC is the second main cause of cancer-related death with an estimated 498,000 deaths occur annually 3. In spite of the improvements in diagnosis and treatment 4, currently, the long-term survival rate for a large number of GC patients is still dismal, with a 5-year survival rate of less than 20% 5. Most patients have developed tumor metastasis when they are diagnosed with GC, and the high tumor metastasis rate leads to unfavorable survival outcome in patients with GC 6. Identifying a biomarker for early diagnosis and clinical outcome prediction is important to understand the development of GC and further improve the prognosis in patients with GC.

Antigen Ki-67, also known as MKI67 (antigen identified by monoclonal antibody Ki-67), is a cell proliferation-related protein that encoded by gene MKI67. Ki-67/MKI67 exists in G1, S, G2 and mitosis phases of cell cycle 7 and can be put into clinical practice by acting as an index to evaluate cell proliferation by immunohistochemical staining 8. A large number of studies have reported the clinical and prognostic value of Ki-67/MKI67 in various malignancies, such as breast cancer 9, cervical cancer 10, glioma 11, colorectal cancer 12 and hepatocellular carcinoma 13. Our research group previously conducted systematic meta-analyses to explore the prognostic role of Ki-67/MKI67 in lung cancer 14, hepatocellular carcinoma 13, cervical cancer 15, glioma 16 and colorectal cancer 17 and found that Ki-67/MIB-1 could be an independent prognostic biomarker in these types of carcinoma.

Although two previous meta-analyses have reported the potential of Ki-67/MKI67 as a predictive biomarker in patients with GC 18, 19, studies based on more cases and stronger evidence are still needed to corroborate the prognostic and clinicopathological role of Ki-67/MKI67 for GC patients. Thus, we conducted a comprehensive literature search and performed a systematic analysis on the prognostic value and clinical significance of Ki-67/MKI67 in GC. Simultaneously, we investigated the underling action mechanism by which Ki-67/MKI67 affects cell proliferation and promotes tumor development by performing bioinformatics analysis on genes co-expressed with MKI67.

## Methods

### Publication identification and searching strategy

A comprehensive literature search via PubMed, PMC, Science Direct, Web of Science, Wiley Online, Chinese National Knowledge Infrastructure (CNKI), Chinese WanFang database, Chinese Chongqing VIP and Embase was carried out up to March 5, 2019 based on the following searching terms: (Ki-67 OR Ki67 OR MKI67 OR MIB-1 OR proliferative index OR proliferative activity OR mitotic index OR mitotic activity OR mitotic figure OR labeling index OR mitotic count OR proliferative marker) AND (prognos* OR surviv* OR predict* OR follow-up OR followed-up OR mortality OR outcome OR diagnosis OR diagnostic OR detect) AND (gastric OR stomach OR tummy) AND (cancer OR carcinoma OR tumo* OR neoplas* OR malignan*).

### Eligibility criteria for inclusion and exclusion

Two investigators evaluated all of the acquired records independently using the criteria listed below: (1) All patients were distinctly diagnosed with gastric cancer by pathology; (2) The studies were written in English or Chinese; (3) The studies investigated the expression level of Ki-67/MKI67 in gastric cancer and adjacent non-cancerous tissues; (3) The studies reported the relationships of Ki-67/MKI67 expression with prognosis or clinicopathological parameters in gastric cancer patients; (4) The studies provided enough information to estimate the hazard ratios (HRs), odds ratios (ORs) and their 95% confidence intervals (CIs); (5) The latest and most complete study was included when the same patients were reported in different publications; (6) The studies must be performed in humans.

The following publications were excluded: conference abstracts, reviews, meta-analyses, case reports, expert opinions, studies not written in English or Chinese, and studies without sufficient data to calculate HRs, ORs and their 95% CIs. Because of the heterogeneous expression of Ki-67 in GC, tissue microarrays (TMAs) carry the risk of sampling error due to an underestimation and non-representative evaluation of Ki-67 in GC 20. Therefore, we excluded studies using TMAs.

### Data extraction

Two researchers independently performed the data collection from all of the enrolled studies, with any discrepancies settled by discussion with a third reviewer. The relevant information covered the following details: first author and publication year, region, cut-off value, antibody, quality score, statistical method, and HR with corresponding 95% CI.

### Quality assessment

Two investigators independently read each enrolled prognosis-related record and scored it using the Newcastle-Ottawa scale (NOS) 21. Each study was assessed based on the following three perspectives: selection, comparability and outcome. The score ranged from 0 to 9, and high scores mean high quality of the included records. Additionally, the Quality Assessment of Diagnostic Accuracy Studies-2 (QADAS-2) tool was used to assess the quality of diagnostic accuracy studies 22. The risk of bias and applicability of individual studies were judged as “low”, “high” or “unclear” based on the following four critical domains: patient selection, index test, reference standard, and flow and timing. Both reviewers compared their judgments and achieved a consensus by discussing in conference if the results were controversial.

### Statistical analysis

To determine the ability of Ki-67/MKI67 in discriminating tumor from non-tumorous gastric tissues, we performed summary receiver operating characteristic curve (SROC) analysis and calculated the area under the curve (AUC) value, the diagnostic OR and the corresponding sensitivity and specificity by using Meta-DISc 23.

The HRs and 95% CIs were combined to measure the effects of Ki-67/MKI67 expression on overall survival (OS) and disease-free survival (DFS). We directly extracted the HRs and 95% CIs from the original studies if the survival data were provided; otherwise, we calculated them by using Engauge Digitizer Version 4.1 on the basis of the Kaplan-Meier survival curves. Three investigators read the survival curves independently to improve the precision in the extraction of HRs and 95% CIs. Then we combined the individual HRs into a pooled HR by using Stata 12.0 (StataCorp, College Station, TX, USA) to investigate the correlation between Ki-67/MKI67 expression and prognosis in patients with GC. Subgroup analyses were conducted to determine whether different regions, antibodies, cut-off values and statistical methods resulted in the differences of the overall results. An observed HR>1 and it's 95% CI not crossing 1 indicated that a high expression of Ki-67/MKI67 predicted a poor outcome in gastric cancer patients.

The ORs and 95% CIs were calculated to determine the relationships between Ki-67/MKI67 expression and clinicopathological characteristics, such as gender (female versus male), TNM stage (stage III/IV versus stage I/II), tumor differentiation (poor differentiation versus well/moderate differentiation), lymph node metastasis (yes versus no), distant metastasis (yes versus no), invasion depth (T3/T4 versus Tis/T1/T2), Lauren's classification (diffuse versus intestinal) and vascular invasion (yes versus no). The impacts of Ki-67/MKI67 on clinicopathological parameters were considered statistically significant if the 95% CI did not cross 1. The data calculations were conducted by using Stata 12.0.

To evaluate the stability of the pooled results, we conducted sensitivity analysis by excluding individual studies successively. The χ^2^ test (Chi squared test; Chi^2^) and the I^2^ test (Higgins I-squared test; I^2^) were used to analyze the heterogeneity among studies. If an inter-study heterogeneity existed (P<0.05 and/or I^2^≥50%), a random-effects model was applied; otherwise, a fixed-effects model was chosen. Begg's funnel plot and Egger's test were used to assess the publication bias of the eligible studies. All P<0.05 (tested by two-sided) were considered statistically significant.

### Bioinformatics analysis of genes co-expressed with MKI67

Genes co-expressed with MKI67 were collected from cBioPortal (http://www.cbioportal.org/) online website. Gene Ontology (GO) and Kyoto Encyclopedia of Genes and Genomes (KEGG) pathway analyses were performed with the DAVID (https://david.ncifcrf.gov/) online tool. Protein-protein interaction (PPI) network wwas constructed with the STRING (http://string-db.org) database.

## Results

### Studies selection and characteristics

**Fig 1:**
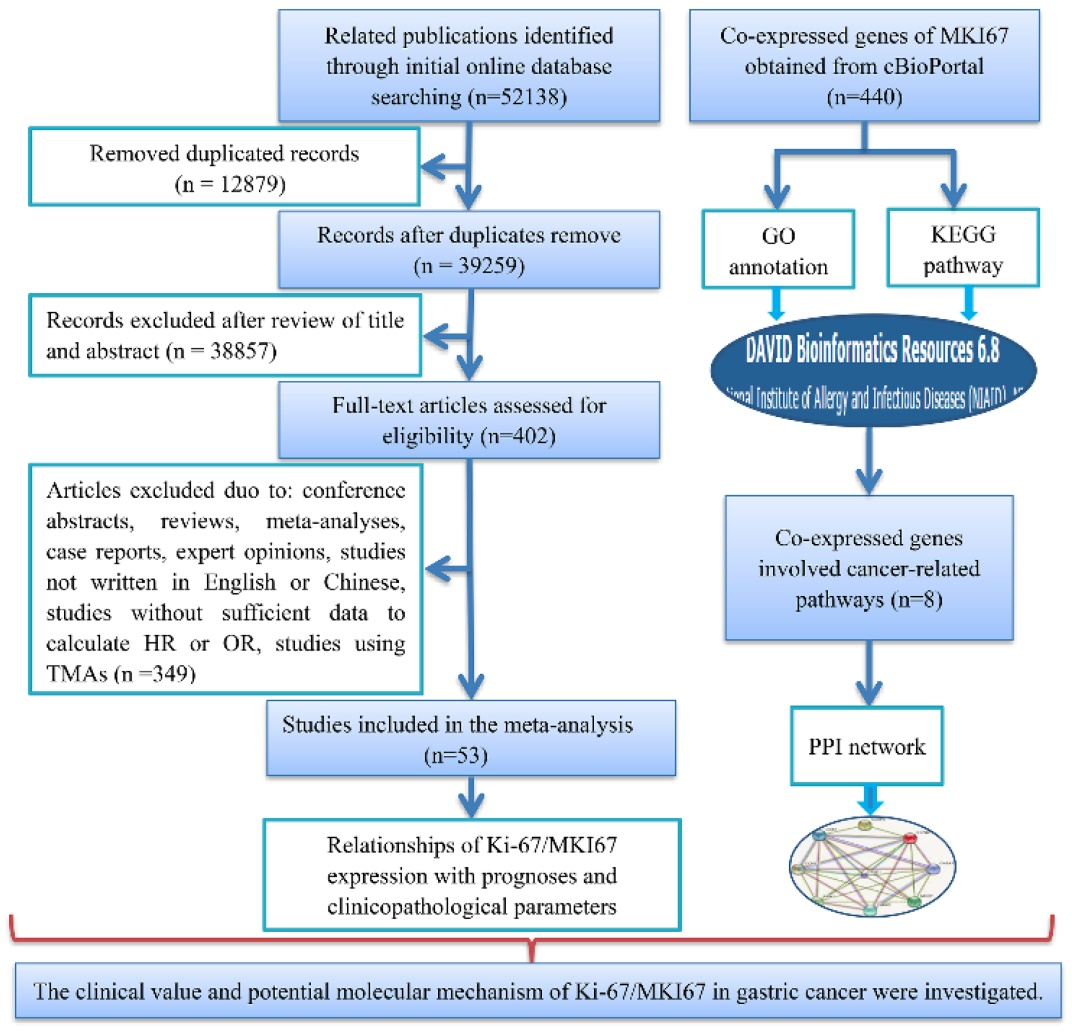
** Flow chart of the overall design of the present study.** TMA: tissue microarray; HR: hazard ratio; OR: odds ratio; GO: Gene Ontology; KEGG: Kyoto Encyclopedia of Genes and Genomes; PPI: Protein-protein interaction.

A total of 53 studies with 7078 patients were included in accordance with our strict inclusion and exclusion criteria (Fig. 1). The main details of the selected studies are concluded in Table 1. All of the studies were retrospective. Of all of the studies, 24 were performed in China 24-47, 12 in Japan 48-59, 3 in Greece 60-62 , 2 in Italy 63, 64, 2 in Poland 65, 66, 1 in Korea 67, 1in Germany 68, 2 in Egypt 69, 70, 1 in Finland 71, 1 in Timisoara 72, 1 in Turkey 73, 1 in Sultanate of Oman 74, 1 in Tunisia 75 and 1 in Saudi Arabia 76. A total of 10 publications were written in Chinese and 43 articles were written in English. The number of patients in each study ranged from 40 to 693. Immunohistochemistry (IHC) method was applied to detect the Ki-67 protein expression: MIB-1 was utilized in 24 studies and anti-Ki-67 antibody was used in 29 studies. Of the 53 included records, 3 records compared the expression of Ki-67/MKI67 in gastric cancerous and adjacent non-cancerous tissues, 44 records reported the prognostic value of Ki-67/MKI67 in GC, and 26 records assessed the relationships between Ki-67/MKI67 and clinicopathological parameters. Among the 44 prognosis-related studies, 20 studies provided HRs and corresponding 95% CIs for OS and DFS directly, and 24 studies only provided Kaplan-Meier survival curves. The methodological quality scores assessed by the NOS scale varied from 6 to 9.

### Expression level of Ki-67/MKI67 in GC

**Fig 2:**
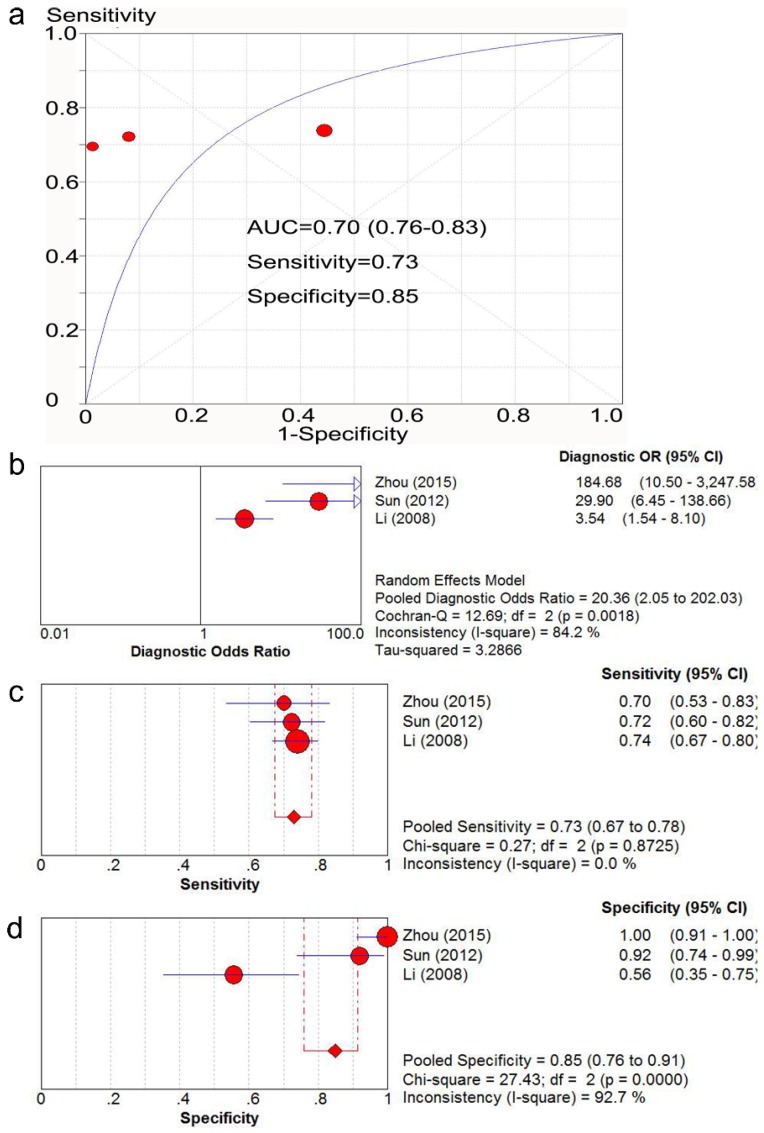
** SROC analysis of the ability of Ki-67/MKI67 to distinguish gastric cancer patients from normal controls.** The AUC value (a), the diagnostic OR (b), the corresponding sensitivity (c) and specificity (d) were calculated using Meta-DISc software. SROC: summary receiver operating characteristic curve. AUC: area under the value; OR: odds ratio; CI: confidence interval.

Three studies 35, 40, 44 with 384 patients provided sufficient data to compare the expression of Ki-67/MKI67 in gastric cancerous and adjacent non-cancerous tissues. The potential risk of bias and applicability of the three studies are shown in Table 2. The AUC value of SROC analysis was 0.80 (95% CI: 0.76-0.83; Fig. 2a), the diagnostic OR was 20.36 (95% CI: 2.05-202.03; Fig. 2b), the corresponding sensitivity and specificity were 0.73 (95% CI: 0.67-0.78; Fig. 2c) and 0.85 (95% CI: 0.76-0.91; Fig. 2d), respectively.

### Prognostic value of Ki-67/MKI67 in GC

**Fig 3:**
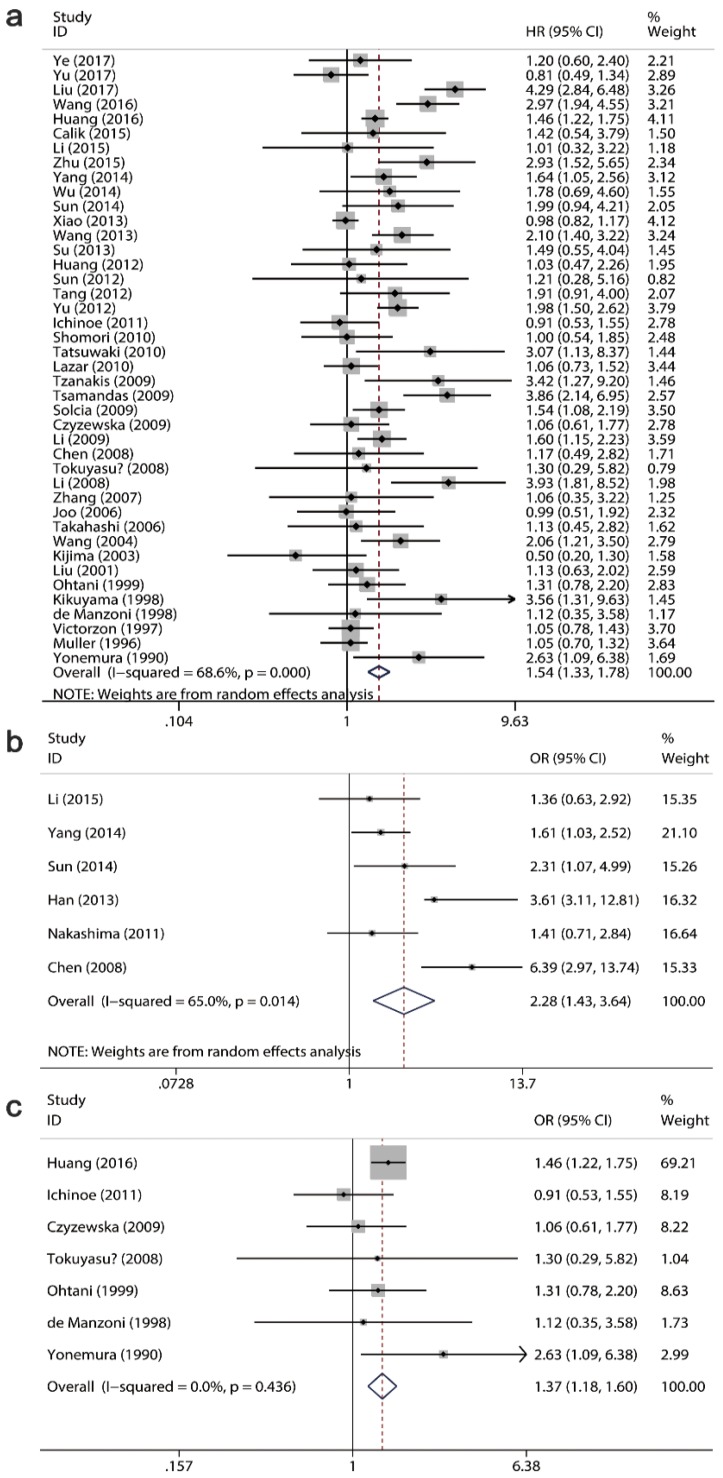
** Forest plots of the pooled harzard ratio (HR) for overall survival (OS) and disease-free survival (DFS).** HR>1 indicates a worse OS for the group with an elevated Ki-67/MKI67 expression. (a) A high expression of Ki-67/MKI67 indicated a poor OS in patients with gastric cancer (GC). (b) A high expression of Ki-67/MKI67 indicated a poor DFS in patients with GC. (c) A high expression of Ki-67/MKI67 indicated a poor OS in patients with advanced GC.

A total 42 studies with 6337 patients assessed the relationship of Ki-67/MKI67 expression with OS, while 6 studies with 564 cases evaluated the association between Ki-67/MKI67 expression and DFS. Among the 42 OS-related studies, 7 studies reported the prognostic role of Ki-67/MKI67 in advanced GC. The pooled HRs from a random-effects model indicated that an elevated expression of Ki-67/MKI67 predicted a poor OS (HR: 1.54, 95% CI: 1.33-1.78, P<0.0001; I^2^=69.6, P<0.0001; Fig. 3a) and DFS (HR: 2.28, 95% CI: 1.43-3.64, P<0.0001; I^2^=65, P=0.014; Fig. 3b) in patients with gastric cancer. Additionally, the combined HR from a fixed-effects model showed that a high Ki-67/MKI67 expression was also significantly connected with OS in patients with advanced GC (HR: 1.37, 95% CI: 1.18-1.60, P<0.0001; I^2^=0, P=0.436; Fig. 3c).

Then we conducted subgroup analyses to determine whether different regions, antibodies, cut-off values and statistical methods resulted in the alterations of the pooled results (Table 3). Subgroup analysis on regions for OS suggested that a high Ki-67/MKI67 expression predicted a worse survival outcome both in Asian (HR: 1.58, 95% CI: 1.33-1.88, P<0.0001) and non-Asian populations (HR: 1.40, 95% CI: 1.06-1.85, P=0.02). For DFS, the pooled HR in Asian subgroup was 2.52 (95% CI=1.48-4.32, P<0.0001). There was only one study evaluating the relationship between Ki-67/MKI67 expression and DFS in non-Asian subgroup, and the HR was 1.41 (95% CI: 0.71-2.81, P=0.329), respectively. Subgroup analysis on antibodies showed that a highly expressed Ki-67/MKI67 indicated poor OS both in MIB-1 group (HR=1.49, 95% CI=1.21-1.84, P<0.0001) and anti-Ki-67 group (HR=1.56, 95% CI=1.27-1.92, P<0.0001). For DFS, the pooled HRs in MIB-1 and anti-Ki-67 groups were 2.97 (95% CI=0.68-13.07, P=0.149) and 2.00 (95% CI=1.30-3.00, P=0.001). We stratified all of the included studies into high and low cut-off value groups according to the cut-off values of 0.2, 0.25, 0.3, 0.4 and 0.45 to identify a cut-off value of Ki-67/MKI67 expression that could be strongly related to survival expectancy (data not shown). We found that the group with a cut-off value less than 0.25 showed the highest HR, and the group with a cut-off value more than 0.25 showed the lowest HR. For OS, the HRs in high and low cut-off value groups were 1.38 (95% CI: 1.17-1.63, P<0.0001) and 1.77 (95% CI: 1.37-2.28, P<0.0001), respectively. Two studies did not provide the cut-off value of Ki-67/MKI67 expression, and the pooled HR of the two articles was 2.00 (95% CI: 1.36-2.93, P<0.0001). For DFS, the overall results suggested that highly expressed Ki-67/MKI67 was related to worse survival outcome both in high cut-off value group (HR: 1.51, 95% CI: 1.08-2.11, P=0.017) and low cut-off value group (HR: 3.76, 95% CI: 2.14-6.60, P<0.0001).

Subgroup analysis on the type of statistical methods applied to extract HR was also conducted. For OS, the pooled HRs in multivariate analysis, univariate analysis and survival curve subgroups were 1.62 (95% CI=1.24-2.12, P<0.0001), 1.59 (95% CI=0.90-2.80, P=0.111) and 1.46 (95% CI=1.23-1.74, P<0.0001), respectively. For DFS, the combined HR in survival curve subgroup was 2.92 (95% CI=1.41-5.97, P<0.0001). While there was only one study evaluating the relationship between Ki-67/MKI67 expression and DFS in multivariate analysis and univariate analysis subgroups, and the HRs were 1.36 (95% CI=0.63-2.92, P=0.433) and 1.41 (95% CI: 0.71-2.81, P=0.329), respectively.

### Clinicopathological significance of Ki-67/MKI67 for GC patients

**Fig 4:**
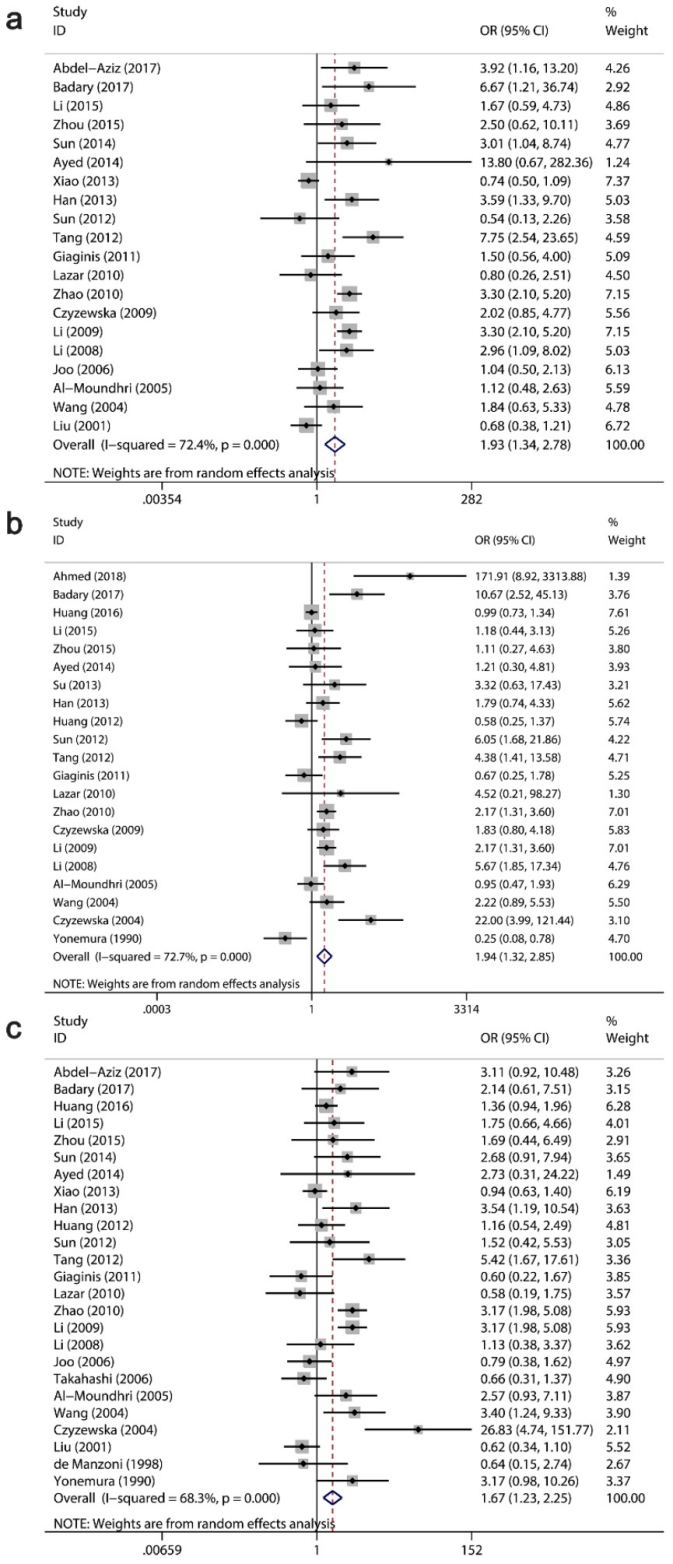
** Relationships between Ki-67/MKI67 expression and TNM stage, tumor differentiation and lymph node metastasis.** (a) TNM stage (stage III/IV *versus* stage I/II). (b) Tumor differentiation (poor *versus* well/moderate). (c) Lymph node metastasis (yes *versus* no).

**Fig 5:**
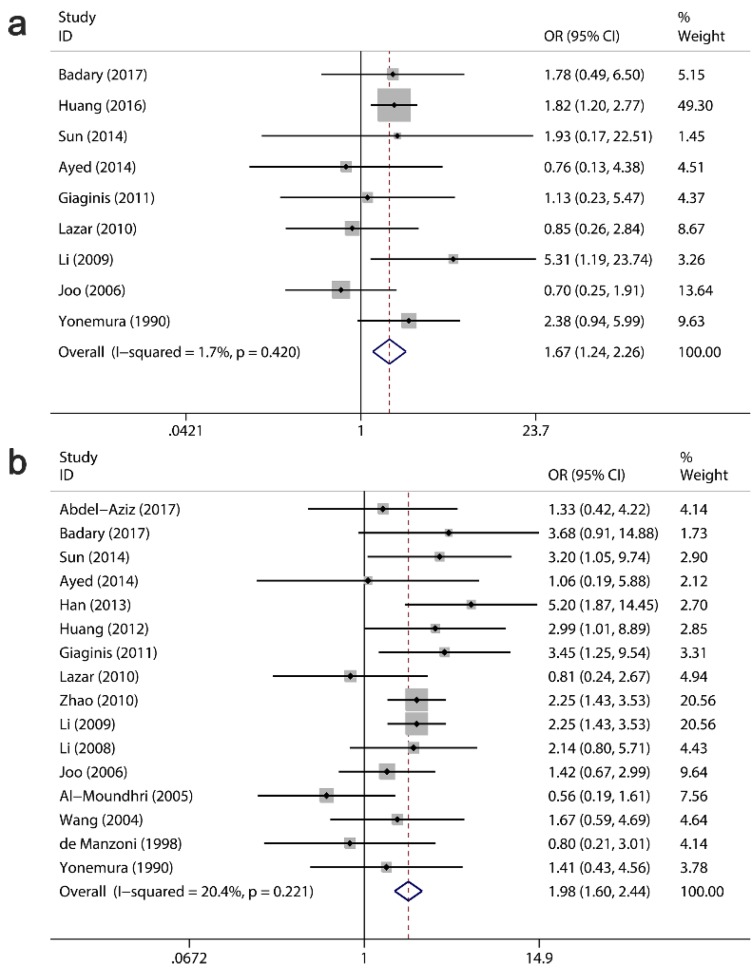
** Relationships between Ki-67/MKI67 expression and distant metastasis, invasion depth and Lauren's classification.** (a) Distant metastasis (yes *versus* no). (b) Tumor invasion depth (T3/T4 *versus* Tis/T1/T2).

The clinicopathological value of Ki-67/MKI67 expression for GC patients was assessed (Table 4). The combined ORs from a random-effects model suggested that an up-regulation of Ki-67/MKI67 was related to TNM stage (stage III/IV versus stage I/II: OR=1.93, 95% CI=1.34-2.78, P<0.0001; I²=72.4, P<0.0001; Fig. 4a), tumor differentiation (poor versus well/moderate: OR=1.94, 95% CI=1.32-2.85, P =0.001; I²=72.4, P<0.0001; Fig. 4b) and lymph node metastasis (yes versus no: OR=1.67, 95% CI=1.23-2.25, P=0.001; I²=68.3, P<0.0001; Fig. 4c). The pooled ORs from a fixed-effects model showed that an increased expression of Ki-67/MKI67 was correlated with distant metastasis (yes versus no: OR=1.67, 95% CI=1.24-2.26, P=0.001; I²=1.7, P=0.42; Fig. 5a) and tumor invasion depth (T3/T4 versus Tis/T1/T2: OR=1.98, 95% CI=1.60-2.44, P<0.0001; I²=20.4, P=0.221; Fig. 5b). No statistically significant correlations were found of Ki-67/MKI67 expression with gender (female versus male: OR=1.00, 95% CI=0.84-1.19, P=0.986; I²=0, P=0.541), Lauren's classification (intestinal versus diffuse: OR=1.21, 95% CI=0.97-1.53, P=0.096; I²=46.3, P=0.053) and vascular invasion (yes versus no: OR=1.66, 95% CI=0.70-3.95, P=0.253; I²=72.2, P=0.006).

### Sensitivity analysis

**Fig 6:**
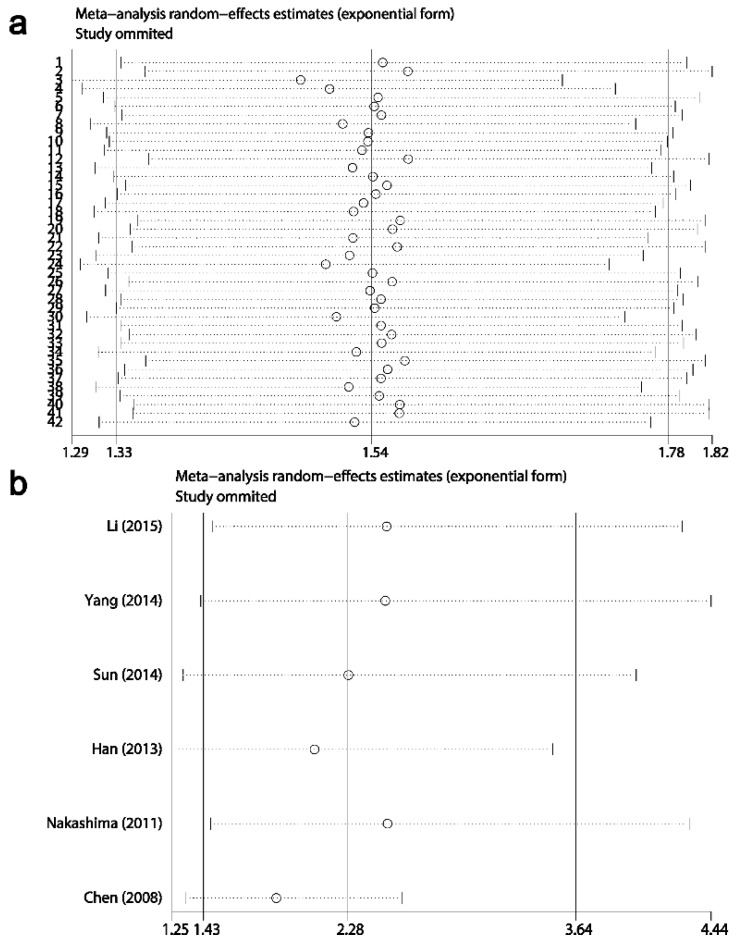
Result of sensitivity analysis from a random-effects model for **overall survival** (a) and disease-free survival (b). Three vertical lines indicated the pooled hazard ratio (HR) with 95% confidence interval (CI) calculated from all included studies. Each dotted horizontal line belongs to an independent meta-analysis. And the middle circle and two sides' short vertical lines represents the pooled HR and its 95% CI corresponding to the successive exclusion of each study.

**Fig 7:**
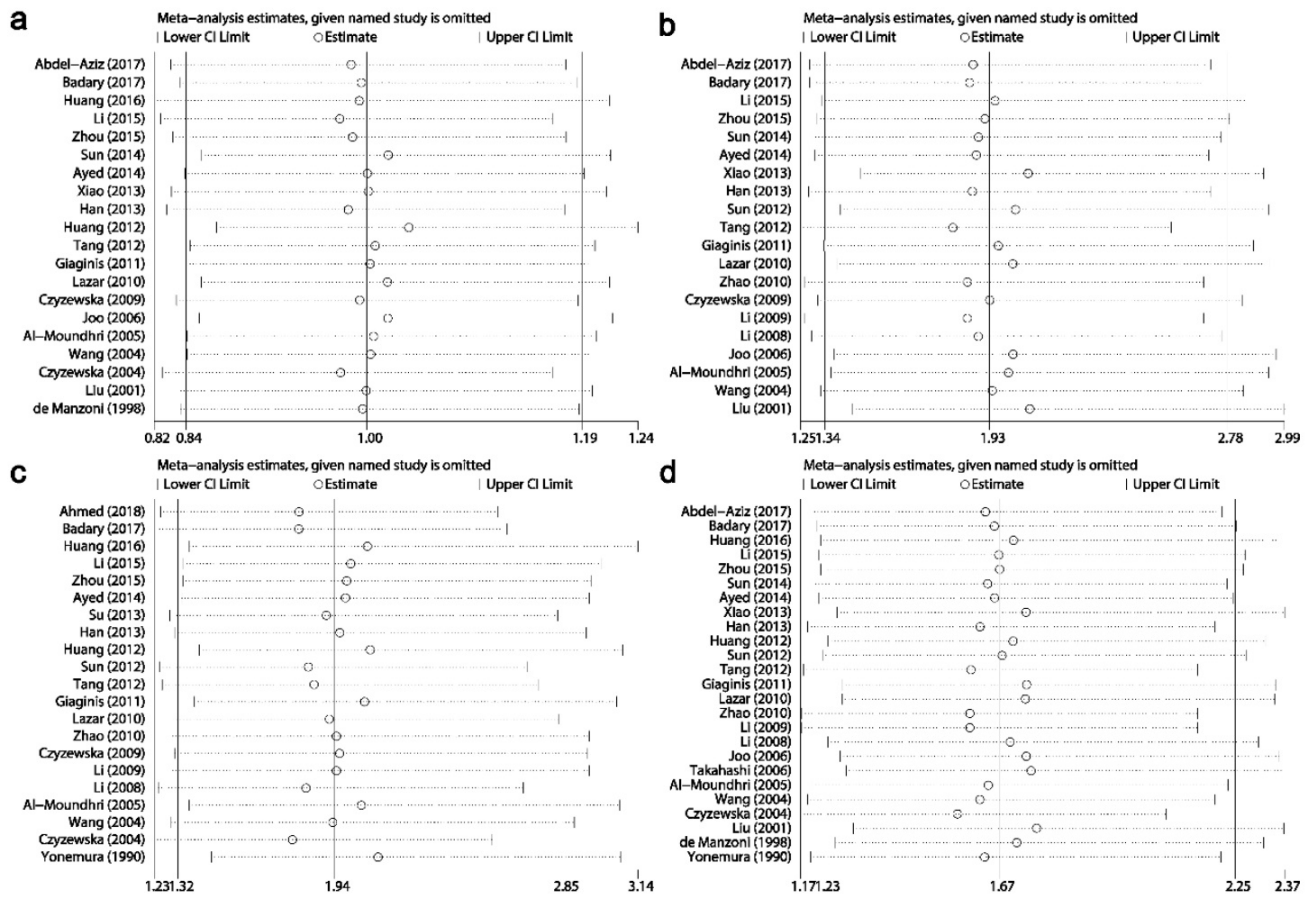
Results of sensitivity analysis for relationships between Ki-67/MKI67 expression and clinicopathological characteristics. (a) Gender. (b) TNM stage. (c) Tumor differentiation. (d) Lymph node metastasis.

**Fig 8:**
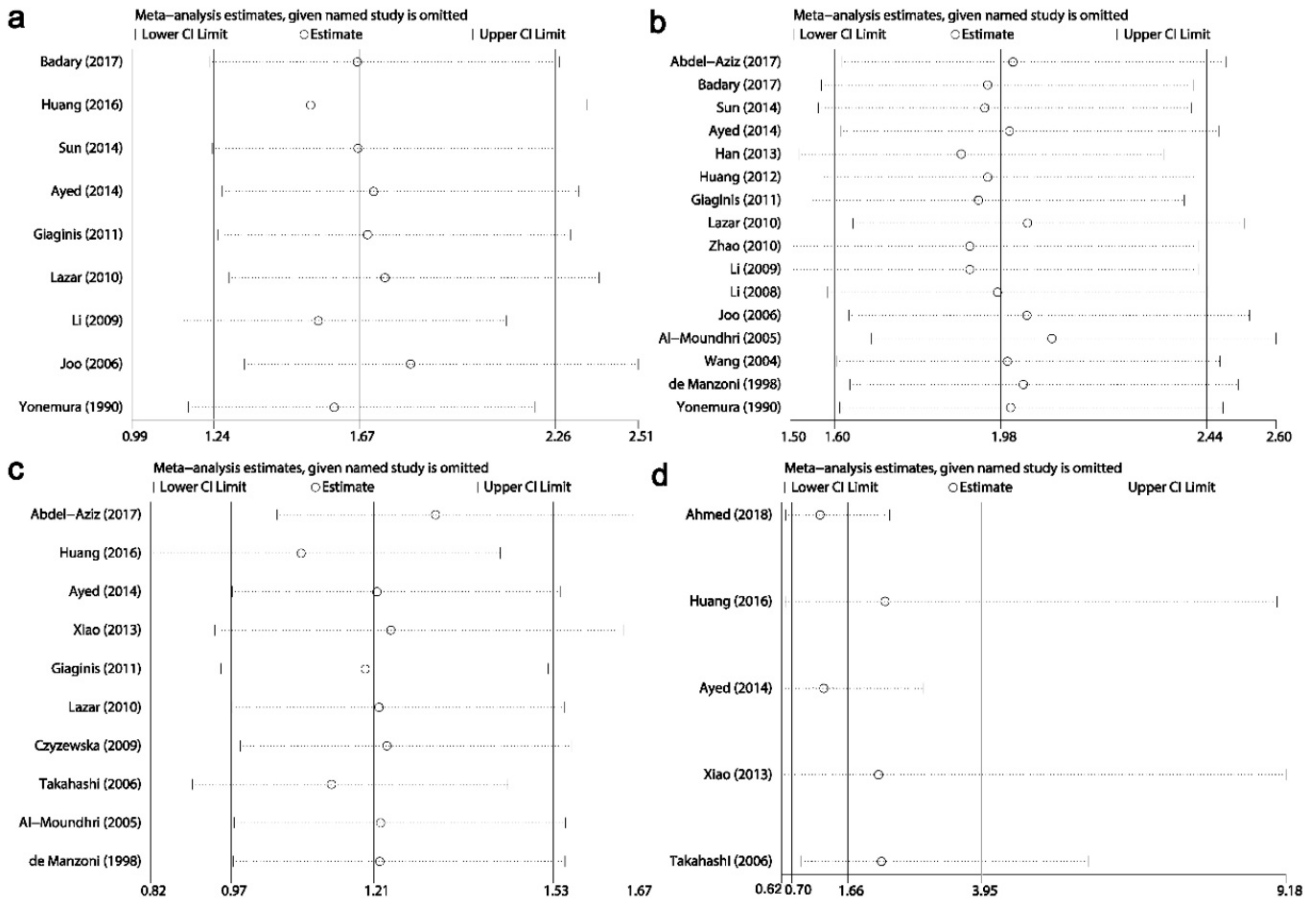
Results of sensitivity analysis for relationships between Ki-67/MKI67 expression and clinicopathological characteristics. (a) Distant metastasis. (b) Tumor invasion depth. (c) Lauren's classification. (d) Vascular invasion.

Results of sensitivity analysis showed that the combined HRs and ORs corresponding to the successive exclusion of individual reports were not significantly altered, indicating that the pooled results of the current meta-analysis were stable (Figs. 6-8).

### Publication bias

**Fig 9:**
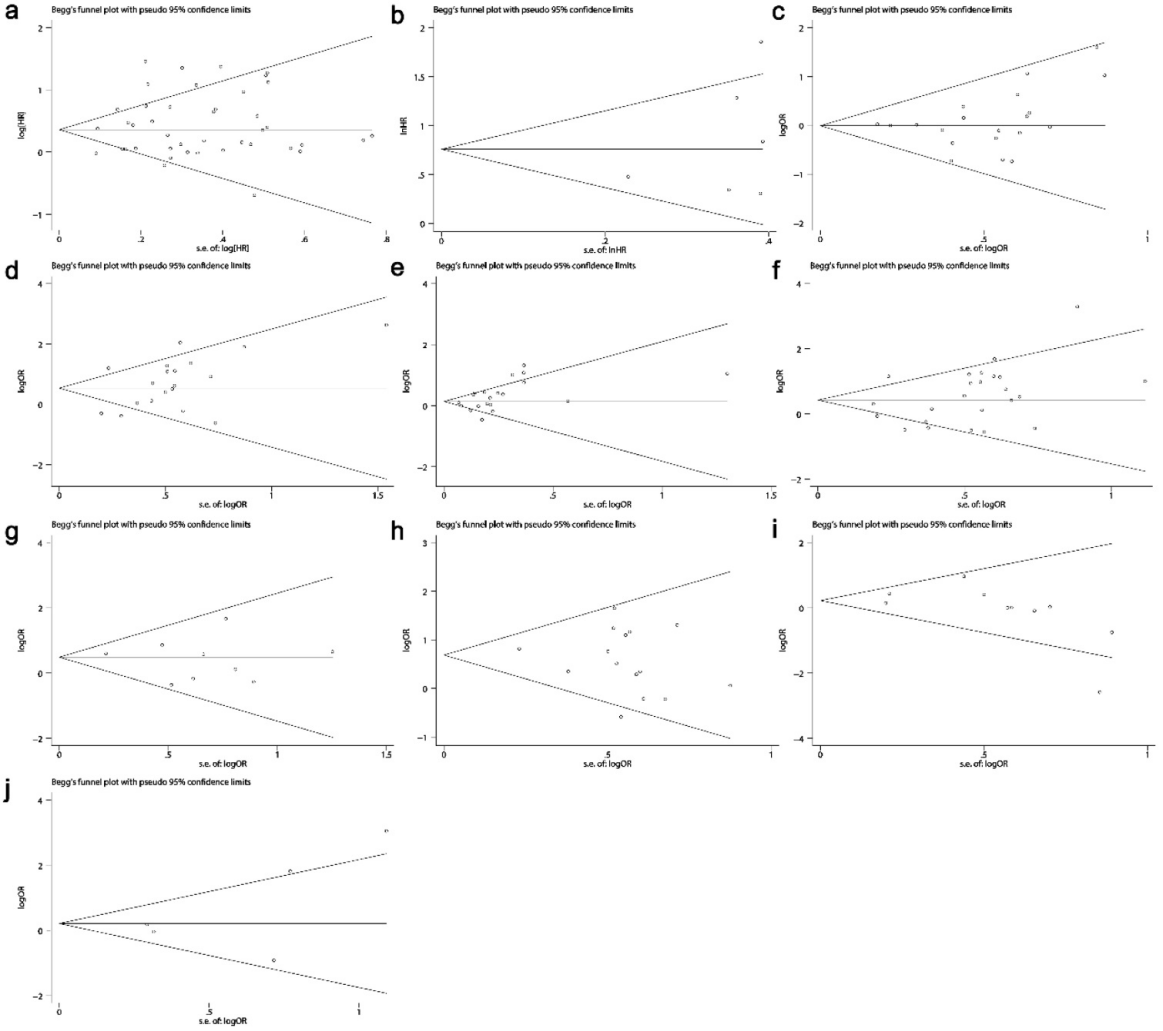
Funnel plots evaluating potential publication bias among the included studies. (a) Overall survival. (b) Disease-free survival. (c) Gender. (d) TNM stage. (e) Tumor differentiation. (f) Lymph node metastasis. (g) Distant metastasis. (h) Invasion depth. (i) Lauren's classification. (j) Vascular invasion. The circles represent an individual study enrolled in the present meta-analysis.

The Begg's funnel plots are presented in Fig. 9. No publication bias existed in all situations (Table 3 and Table 4).

### Potential action mechanism of MKI67 in GC

**Fig 10:**
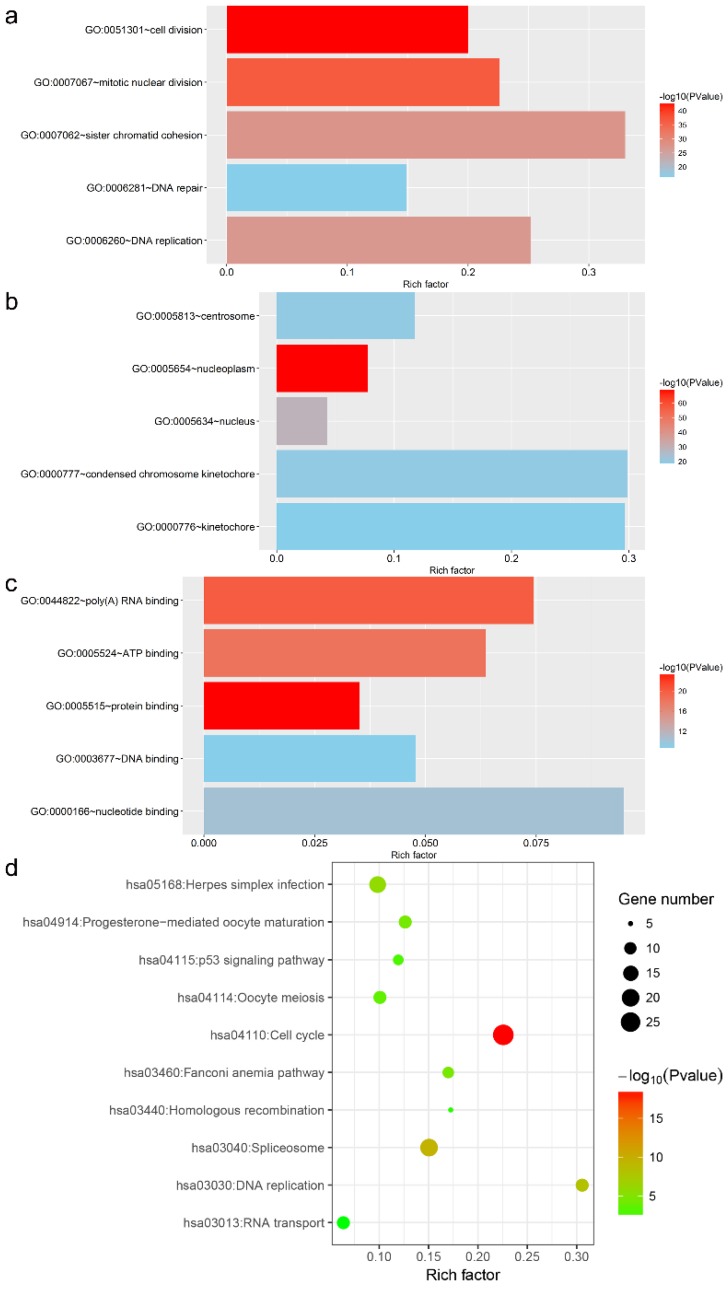
Top five Gene Ontology functional annotations and top ten Kyoto Encyclopedia of Genes and Genomes pathways of co-expressed genes of MKI67.

In total, 440 genes co-expressed with MKI67 in GC were collected for GO, KEGG pathway and PPI network analyses. The top five GO functional annotations and top ten KEGG pathways are presented in Fig. 10. The results of GO enrichment analyses indicated that the main biological functions of MKI67 were related to cell division, nucleoplasm and protein binding. The results of KEGG pathway analysis revealed that these co-expressed genes were involved in P53 signaling pathway.

**Fig 11:**
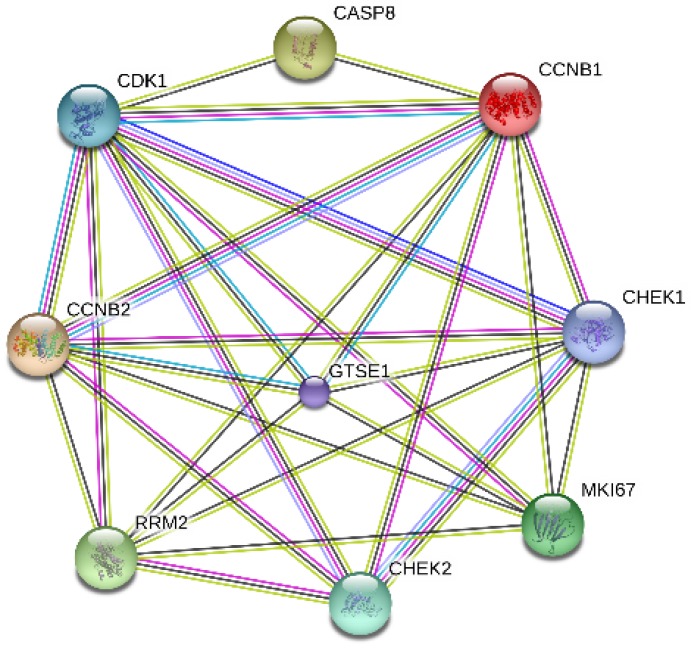
Interactions between MKI67 and eight genes related to P53 signaling pathway.

MKI67 and its co-expressed genes that involved in P53 signaling pathway were selected for PPI network construction. The following 8 genes were chosen: cyclin B1 (CCNB1), cyclin-dependent kinase 1 (CDK1), cyclin B1 (CCNB2), ribonucleotide reductase M2 (RRM2), caspase 8 (CASP8), checkpoint kinase 1(CHEK1), checkpoint kinase 2 (CHEK2) and G-2 and S-phase expressed 1 (GTSE1). According to our results, MKI67 directly interacted with genes RRM2, CCNB2, GTSE1, CDK1, CCNB1 and CHEK1 (Fig. 11).

## Discussion

In the present study, we firstly investigated the ability of Ki-67/MKI67 in discriminating tumor from normal controls. We found that an over-expressed Ki-67/MKI67 could differentiate gastric cancer patients from normal subjects. However, the result should be interpreted with caution because only three relevant studies were included and an inter-study heterogeneity among the three studies was discovered. Further researches with larger sample scales are necessary to validate our result.

A number of studies have indicated that Ki-67/MKI67 is a predictive marker for tumor deterioration and its expression shows an association with unfavorable prognosis and malignant clinical phenotypes in various cancers, such as bladder cancer 77, non-small cell lung cancer 78, hepatocellular carcinoma 13, cervical cancer 10 and glioma 16. The clinical and prognostic value of Ki-67/MKI67 in GC has also been investigated previously. Luo et al. 19 has conducted a meta-analysis based on 29 studies and demonstrated that a high Ki-67/MKI67 expression predicted poor OS and DFS in patients with GC. The authors also discovered that an increased Ki-67/MKI67 expression was related to Lauren's classification and tumor size. Liu et al. 18 also performed a meta-analysis and found that a highly expressed Ki-67/MKI67 predicted an inferior OS in 3825 patients with GC. Compare with Luo's and Liu's studies, our study included much more samples, resulting in more persuasive and stronger conclusions. A total of 53 studies with 7078 patients were enrolled in our meta-analysis. We demonstrated the prognostic and clinicopathological value of Ki-67/MKI67 for GC patients. Especially, we revealed the potential of Ki-67/MKI67 as a prognostic biomarker in advanced gastric cancer patients.

Metastasis is the most important cause of cancer-related death in patients with GC. Identifying a marker to predict the presence of metastasis and stratify patients into different risk groups is helpful in improving the prognosis of GC patients. Here, an up-regulation of Ki-67/MKI67 was discovered in patients with advanced TNM stage, poor tumor differentiation, serosa and neighboring organs invasion, lymph node metastasis and distant metastasis, suggesting that Ki-67/MKI67 could function as an indicator to predict gastric cancer progression and identify high-risk patients, thereby optimizing individual treatment management and improving the prognosis of patients with GC.

Ki-67/MKI67 is a well-known cell cycle-related protein. A recent study has corroborated that specific Ki-67/MKI67 splice variants promote cancer progression by influencing cell cycle 79. It is reported that P53 exert inhibitory effect on Ki-67 promoter by regulating P53- and SP1-dependent pathways 80. Interesting, we also demonstrated the close link between Ki-67/MKI67 and P53 signaling pathway. We found that MKI67 interacted with genes RRM2, CCNB2, GTSE1, CDK1, CCNB1 and CHEK1, which are critical molecules those are involved in P53 signaling network and related to cell cycle progression. Based on previous studies and our evidence, we hypothesize that MKI67 may promote the initiation and progression of GC via interacting with P53 signaling pathway. Further *in vitro* and *in vivo* experiments are necessary to confirm our conclusions.

There were still several deficiencies and limitations in our study. First, an obvious inter-study heterogeneity among the included studies was discovered in the present meta-analysis. There were no unified standards for the antibody concentration, IHC staining degree and cut-off value in different studies, which may be part of the reasons for the heterogeneity. Additionally, the differences in clinicopathological characteristics such as age, gender, tumor stage may also lead to the heterogeneity. A random-effects model was applied to eliminate the effect of the heterogeneity on our results. Remarkably, although the heterogeneity existed in our study, the results of sensitivity analyses indicated that no individual researches influenced the pooled HRs and ORs. Second, in 24 studies, HRs with 95% CIs had to be calculated from the Kaplan-Meier survival curves, which may decrease the reliability and accuracy of our meta-analysis. To reduce the deviation, three researchers independently extracted HRs from the Kaplan-Meier curves; the similarity between the copy curves drawn by the extracted data and original curves was the standard to identify which HRs could be used. Third, our meta-analysis depended on fully published studies written in English or Chinese, which may contribute to potential language bias. In addition, studies with positive results are more likely to be published in magazines. Thus, we should not ignore the potential bias even though the results from Begg's and Egger's tests showed no publication bias in the present study.

## Conclusions

Our meta-analysis provides evidence that Ki-67/MKI67 not only could be a potential prognostic biomarker in clinic for GC patients but also could be an indicator to predict GC progression and to identify high-risk cases, thereby optimizing individual treatment management and improving the prognosis of GC patients. More interesting, we also found that gene MKI67 probably promoted the occurrence and development of GC by influencing P53 signaling pathway. Further well-designed studies are required to validate our conclusions.

## Figures and Tables

**Table 1:** Main characteristics of the 53 studies included in the meta-analysis

ID	First author (publication year)	Regions	Cut-off value	Antibody	Statistical method	HR (95% CI)	Quality score
1	Ye (2017)	China	0.25	Anti-Ki-67	Multivariate analysis	OS: 1.197 (0.598-2.397)	7
2	Liu (2017)	China	0.1	Anti-Ki-67	Multivariate analysis	OS: 4.290 (2.839-6.483)	7
3	Yu (2017)	China	0.5	Anti-Ki-67	Univariate analysis	OS: 0.807 (0.487-1.336)	8
4	Wang (2016)	China	0.5	Anti-Ki-67	Univariate analysis	OS: 2.974 (1.944-4.550)	7
5	Huang (2016)	China	0.5	Anti-Ki-67	Multivariate analysis	OS: 1.46 (1.215-1.754)	8
6	Calik (2015)	Turkey	0.1	MIB-1	Survival curve	OS: 1.42 (0.54-3.79)	6
7	Li (2015)	China	0.5	Anti-Ki-67	Survival curve Multivariate analysis	OS: 1.01 (0.32-3.22) DFS: 1.358 (0.632-2.917)	7
8	Zhu (2015)	China	0.5	Anti-Ki-67	Multivariate analysis	OS: 2.927 (1.518-5.646)	8
9	Yang (2014)	China	0.3	Anti-Ki-67	Survival curve	OS: 1.64 (1.05-2.56) DFS: 1.61 (1.03-2.52)	8
10	Wu (2014)	China	0.12	Anti-Ki-67	Survival curve	OS: 1.78 (0.69-4.6)	8
11	Sun (2014)	China	0.05	Anti-Ki-67	Survival curve	OS: 1.99 (0.94-4.21) DFS: 2.31 (1.07-4.99)	8
12	Xiao (2013)	Japan	0.5	Anti-Ki-67	Multivariate analysis	OS: 0.98 (0.82-1.17)	7
13	Wang (2013)	China	NA	Anti-Ki-67	Multivariate analysis	OS: 2.10 (1.40-3.22)	8
14	Su (2013)	China	NA	Anti-Ki-67	Survival curve	OS: 1.49 (0.55-4.04)	7
15	Huang (2012)	China	0.25	Anti-Ki-67	Survival curve	OS: 1.03 (0.47-2.26)	8
16	Sun (2012)	China	0.05	Anti-Ki-67	Survival curve	OS: 1.21 (0.28-5.16)	8
17	Tang (2012)	China	0.1	Anti-Ki-67	Survival curve	OS: 1.91 (0.91-4)	7
18	Yu (2012)	China	0.2	MIB-1	Multivariate analysis	OS: 1.982 (1.5-2.62)	8
19	Ichinoe (2011)	Japan	0.4	MIB-1	Univariate analysis	OS: 0.907 (0.532-1.547)	7
20	Shomori (2010)	Japan	0.45	Anti-Ki-67	Univariate analysis	OS: 1 (0.54-1.85)	9
21	Tatsuwaki (2010)	Japan	0.25	MIB-1	Univariate analysis	OS: 3.07 (1.13-8.37)	8
22	Lazar (2010)	Timisoara	0.45	MIB-1	Survival curve	OS: 1.06 (0.73-1.52)	8
23	Tzanakis (2009)	Greece	0.35	MIB-1	Univariate analysis	OS: 3.42 (1.27-9.2)	8
24	Tsamandas (2009)	Greece	0.05	MIB-1	Survival curve	OS: 3.86 (2.14-6.95)	8
25	Solcia (2009)	Italy	0.4	MIB-1	Multivariate analysis	OS: 1.54 (1.08-2.19)	8
26	Czyzewska (2009)	Poand	0.5	MIB-1	Survival curve	OS: 1.06 (0.61-1.77)	9
27	Li (2009)	China	0.1	Anti-Ki-67	Survival curve	OS: 1.6 (1.15-2.23)	7
28	Tokuyasu (2008)	Japan	0.1	MIB-1	Survival curve	OS: 1.3 (0.29-5.82)	9
29	Chen (2008)	China	0.1	MIB-1	Survival curve	OS: 1.17 (0.49-2.82) DFS: 6.39 (2.97-13.74)	7
30	Li (2008)	China	0.5	Anti-Ki-67	Survival curve	OS: 3.93 (1.81-8.52)	8
31	Zhang (2007)	China	0.1	Anti-Ki-67	Survival curve	OS: 1.06 (0.35-3.22)	7
32	Joo (2006)	Korea	0.5	MIB-1	Survival curve	OS: 0.99 (0.51-1.92)	8
33	Takahashi (2006)	Japan	0.5	MIB-1	Survival curve	OS: 1.13 (0.45-2.82)	8
34	Wang (2004)	China	0.53	Anti-Ki-67	Survival curve	OS: 2.06 (1.21-3.5)	7
35	Kijima (2003)	Japan	0.1	Anti-Ki-67	Multivariate analysis	OS: 0.5 (0.2-1.3)	6
36	Liu (2001)	Japan	0.27	MIB-1	Multivariate analysis	OS: 1.13 (0.63-2.02)	7
37	Ohtani (1999)	Japan	0.36	MIB-1	Multivariate analysis	OS: 1.31 (0.778-2.2)	8
38	Kikuyama (1998)	Japan	0.55	MIB-1	Multivariate analysis	OS: 3.56 (1.31-9.63)	7
39	de Manzoni (1998)	Italy	0.1	Anti-Ki-67	Multivariate analysis	OS: 1.12 (0.35-3.58)	7
40	Victorzon (1997)	Finland	0.3	Anti-Ki-67	Survival curve	OS: 1.05 (0.78-1.43)	9
41	Muller (1996)	Germany	0.53	MIB-1	Survival curve	OS: 1.05 (0.7-1.32)	8
42	Yonemura (1990)	Japan	0.25	MIB-1	Survival curve	OS: 2.63 (1.09-6.38)	6
43	Han (2013)	China	0.1	Anti-Ki-67	Survival curve	DFS: 6.31 (3.11-12.81)	8
44	Nakashima (2011)	Japan	NA	MIB-1	Univariate analysis	DFS: 1.41 (0.714-2.835)	6
45	Ahmed (2018)	Saudi Arabia	0.1	MIB-1	NA	NA	7
46	Abdel-Aziz (2017)	Egypt	0.45	MIB-1	NA	NA	7
47	Badary (2017)	Egypt	NA	Anti-Ki-67	NA	NA	8
48	Zhou (2015)	China	NA	Anti-Ki-67	NA	NA	8
49	Ayed (2014)	Tunisa	0.01	MIB-1	NA	NA	7
50	Giaginis (2011)	Greece	0.5	MIB-1	NA	NA	8
51	Zhao (2010)	China	0.5	Anti-Ki-67	NA	NA	7
52	Al-Moundhri (2005)	Sultanate of Oman	0.25	MIB-1	NA	NA	7
53	Czyzewska (2004)	Poland	NA	Anti-Ki-67	NA	NA	8

NA: data not available; OS: overall survival; DFS: disease-free survival; HR: hazard ratio; CI: confidence interval.

**Table 2:** Potential risk of bias and applicability of the three diagnostic accuracy studies

	Risk of bias				Applicability		
Study	Patient selection	Index test	References standard	Flow and timing	Patient selection	Index test	References standard
Li (2008)	J	?	?	L	J	J	J
Sun (2012)	J	J	?	L	J	J	J
Zhou (2015)	J	L	?	L	J	L	J

J: low risk; L: high risk; ?: unclear risk.

**Table 3:** Results of subgroup analyses for OS and DFS

				Heterogeneity test		Publication bias	
Group	Number of studies	HR (95% CI)	P	I^2^ (%)	P	Begg's P	Egger's P
**OS**	42	1.54 (1.33-1.78)	<0.0001	68.6	<0.0001	0.573	0.19
**Region**							
Asian	33	1.58 (1.33-1.88)	<0.0001	69.3	<0.0001		
Non-Asian	9	1.40 (1.06-1.85)	0.02	65.3	0.003		
**Antibody**							
MIB-1	18	1.49 (1.21-1.84)	<0.0001	56.6	0.002		
Anti-Ki-67	24	1.56 (1.27-1.92)	<0.0001	74.8	<0.0001		
**Cut-off value**							
Low	18	1.77 (1.37-2.28)	<0.0001	56.2	0.002		
High	22	1.38 (1.17-1.63)	<0.0001	67.2	<0.0001		
NA	2	2.00 (1.36-2.93)	<0.0001	0	0.534		
**Statistical methods**							
Multivariate analysis	13	1.62 (1.24-2.12)	<0.0001	82.1	<0.0001		
Univariate analysis	6	1.59 (0.90-2.80)	0.111	80.4	<0.0001		
Survival curve	23	1.46 (1.23-1.74)	<0.0001	41.4	0.021		
**DFS**	6	2.28 (1.43-3.64)	0.001	65	0.014	0.133	0.376
**Regions**							
Asian	5	2.52 (1.48-4.32)	0.001	68.4	0.013		
Non-Asian	1	1.41 (0.71-2.81)	0.329	None	None		
**Antibody**							
MIB-1	2	2.97 (0.68-13.07)	0.149	87.9	0.004		
Anti-Ki-67	4	2.00 (1.30-3.00)	0.001	35.6	0.199		
**Cut-off value**							
Low	3	3.76 (2.14-6.60)	<0.0001	41	0.183		
High	3	1.51 (1.08-2.11)	0.017	0	0.909		
**Statistical methods**							
Multivariate analysis	1	1.36 (0.63-2.92)	0.433	None	None		
Univariate analysis	1	1.41(0.71-2.81)	0.329	None	None		
Survival curve	4	2.92 (1.41-5.97)	0.001	71.6	0.014		

NA: data not available; OS: overall survival; DFS: disease-free survival; HR: hazard ratio; CI: confidence interval.

**Table 4:** Relationships between Ki-67/MKI67 expression and clinicopathological parameters

					Heterogeneity test			Publication bias	
Clinicopathological features	Number of studies	Number of patients	OR (95% CI)	P value	I² (%)	P value	Model	Begg's P	Egger's P
Gender	20	2574	1.00 (0.84-1.19)	0.986	0	0.541	Fixed-effects model	0.074	0.387
TNM Stage	20	2452	1.93 (1.34-2.78)	<0.0001	72.4	<0.0001	Random-effects model	0.27	0.253
Tumor differentiation	21	2669	1.94 (1.32-2.85)	0.001	72.7	<0.0001	Random-effects model	0.08	0.487
Lymph node metastasis	25	3439	1.67 (1.23-2.25)	0.001	68.3	<0.0001	Random-effects model	0.207	0.317
Distant metastasis	9	1557	1.67 (1.24-2.26)	0.001	1.7	0.42	Fixed-effects model	0.99	0.6
Invasion depth	16	1695	1.98 (1.60-2.44)	<0.0001	20.4	0.211	Fixed-effects model	0.322	0.341
Lauren's classification	10	1602	1.21 (0.97-1.53)	0.096	46.3	0.053	Fixed-effects model	0.074	0.155
Vascular invasion	5	1329	1.66 (0.70-3.95)	0.253	72.2	0.006	Random-effects model	0.462	0.31

OR: odds ratio; CI: confidence interval.

## Abbreviations

GC: gastric cancer
MKI67: antigen identified by monoclonal antibody Ki-67
CNKI: Chinese National Knowledge Infrastructure
HR: hazard ration
OR: odds ratio
CI: confidence interval
TMA: tissue microarray
NOS: Newcastle-Ottawa scale
QADAS-2: Quality Assessment of Diagnostic Accuracy Studies-2
SROC: summary receiver operating characteristic curve
AUC: area under the value
OS: overall survival
DFS: disease-free survival
GO: Gene Ontology
KEGG: Kyoto Encyclopedia of Genes and Genomes
PPI: Protein-protein interaction
IHC: immunohistochemistry
